# A missed orthopaedic injury following a seizure: a case report

**DOI:** 10.1186/1752-1947-1-20

**Published:** 2007-05-10

**Authors:** Laurence O'Connor-Read, Benjamin Bloch, Harry Brownlow

**Affiliations:** 1 Royal Berkshire Hospital, Reading, UK; 2 Milton Keynes General Hospital, Milton Keynes, UK

## Abstract

Numerous orthopaedic injuries can follow a seizure and are often diagnosed late. This is the first documented case of a missed bilateral anterior shoulder dislocation following a seizure. The possible reasons for the greater incidence of posterior dislocations are examined and why bilateral anterior dislocations following a seizure are so rare. The article discusses the reasons for the delay and highlights potential pitfalls and learning points for junior emergency department doctors.

## Background

Muscular contractions generated during a seizure can lead to a variety of musculoskeletal injuries. The literature contains descriptions of fractures and dislocations of the shoulder [1-4], femur [5], acetabulum [6] and compression [7] or burst [8] fractures of the vertebrae following a seizure. The incidence of orthopaedic injuries that are missed following a seizure is unknown. Bilateral shoulder dislocations are uncommon, usually presenting as posterior dislocations following epilepsy, electric shock or electroconvulsive therapy [1]. Bilateral anterior dislocations are rare and are usually of traumatic origin [2].

## Case presentation

A twenty five year old man presented to the Emergency Department following an unwitnessed collapse. After playing on his computer for ten hours overnight he got up from his computer at 4 am and lost consciousness without any warning. He was found by his mother and he appeared to be disorientated.

**Figure 1:**
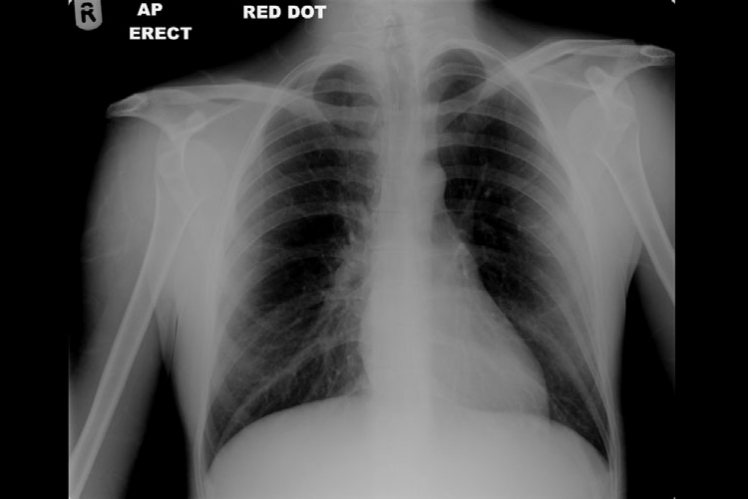
An AP radiograph demonstrating bilateral anterior shoulder dislocations.

The emergency department doctor's examination found a small cut to the nose. The patient was disorientated, exhausted with generalised weakness and subsequent difficulty in moving either arm. Both shoulders were documented as symmetrical with no injury to the soft tissues and grossly neurovascularly intact but were uncomfortable and had limited range of movement. A 'first fit' was diagnosed, bloods were requested and a referral was made to the medical team. The doctor starting the next shift performed a full musculoskeletal examination because of the persisting pain in the shoulders. Radiographs of the shoulders were taken and confirmed bilateral anterior shoulder dislocations (Figure 1). The dislocations were reduced under sedation and the patients' upper limbs were placed in poly-slings. After four weeks of physiotherapy shoulder movements returned to normal.

## Discussion

Following trauma, the shoulder more commonly dislocates anteriorly [9]. As the arm extends and abducts, the coracoacromial arch and rotator cuff cause downward displacement of the humeral head, which is displaced anteriorly by the flexors and external rotators. The posterior dislocations are more common following seizures [1]. The contraction of the relatively weak external rotators of the humerus; infraspinatus, teres minor and the posterior fibres of deltoid are overcome by the more powerful internal rotators; subscapularis, pectoralis major, latissimus dorsi and the anterior fibres of deltoid. The resultant adduction and internal rotation is usually sufficient to cause posterior glenohumeral dislocation.

The bilateral anterior shoulder dislocations following a seizure may occur from the trauma of the shoulders striking the floor after the collapse. On collapsing we rarely see a patient fall in a straight line. A patient would need to land directly forwards or backwards with both his arms abducted and externally rotated to produce the bilateral anterior displacement. The only external injury from our patient was an open wound to his nose, which may suggest that he had fallen straight on to his face in order to sustain this rare presentation.

Cooper in 1839 first reported an association between seizures and posterior shoulder dislocation [10]. In 1902 Mynter first described bilateral posterior shoulder dislocations in a patient following a seizure [11] with numerous cases reported since. Aufranc reported the first bilateral anterior shoulder dislocations following a seizure in 1966 [3]. Only seven further cases have subsequently been reported in the literature [4]. This is the first published case to be missed on initial examination. Because of the absence of any obvious shoulder asymmetry, the patients' generalised weakness and exhaustion, the discomfort and difficulty in moving his arms was initially attributed to a post-ictal state. Full musculoskeletal examinations are not routinely performed following a seizure [12].

The literature suggests that over ten percent of documented bilateral anterior shoulder dislocations following trauma were diagnosed late [2]. As there is a greater awareness of anterior shoulder dislocations following trauma, it would not be unreasonable to assume that there is likely to be a higher incidence of delayed diagnosis of such an injury following a presentation with an indirect complaint, such as a seizure. The unusual presentation combined with the patient's post-ictal discomfort and drowsy state will potentially delay the diagnosis. As this could affect the prognosis, early recognition is vital.

## Conclusion

When the reported rate of late diagnosis is greater than ten percent, in patients with direct trauma [2], the necessity for an accurate examination and imaging in patients complaining of discomfort and weakness in the shoulders following a seizure is evident.

## Competing interests

The author(s) declare that they have no competing interests.

## Authors' contributions

LOCR was involved in the case directly, performed the literature search and drafted part of the manuscript.

BB was involved in the literature review and helped draft part of the manuscript.

HB substantially contributed to revising the manuscript, improving its intellectual content and highlighting its clinical relevance.
